# Fighting the COVID-19 pandemic using the technology-based second-line in Italy and Lombardy: The urgent need of home-based remote monitoring systems to avoid the collapse of the hospital-centred first line

**DOI:** 10.7189/jogh.10.020371

**Published:** 2020-12

**Authors:** Gianluca Castelnuovo, Giada Pietrabissa, Gian Mauro Manzoni, Francesco Sicurello, Italo Zoppis, Enrico Molinari

**Affiliations:** 1 Istituto Auxologico Italiano IRCCS, Psychology Research Laboratory, San Giuseppe Hospital, Verbania, Italy; 2 Catholic University of Milan, Department of Psychology, Milan, Italy; 3 Faculty of Psychology, eCampus University, Novedrate (Como), Italy; 4 AITIM, Italian Association of Telemedicine and Biomedical Informatics, Italy; 5 Department of Computer Science, Università degli Studi di Milano-Bicocca, Milano, Italy

The Italian Health System is quickly empowering the capacity of specific COVID-19 hospital admission (creating new COVID-19 emergency centres-hubs and new intensive care beds in the already existing hospitals) showing a great response in the first-line hospital-based treatment. The major aim of this brief viewpoint is to underline the underestimated role of second line home-based treatment (for positive or suspected patients isolated at home) that could avoid to overwhelm the first line, taking into account the contribution of the necessary social limitations-containment campaign. Particularly the condition of acute respiratory failure could be promptly detected for COVID-19 patients in home isolation without not necessary and potentially dangerous hospital accesses and checks, but using easy-to-use (also for elderly patients and caregivers), comfortable, low-cost, reliable remote monitoring devices, such as connected pulse oximeters that can send real-time vital signs and other key biomedical parameters (especially respiration and blood oxygen levels, body temperature and other parameters) to a hospital-based big data server-repository, where Artificial Intelligence algorithms-based interpretation systems can elaborate these clinical records alerting the physicians only when it is necessary (not too early and not too late) to evaluate if moving a patient from a second to a first line. The experience of biomedical engineering and informatics, strong in chronic care management, could functionally help clinicians in managing many patients and citizens during this strong and long pandemic. It is not possible to postpone the implementation of large remote monitoring programs providing digital devices centrally controlled. We hope that Italian and International scientific and clinical community could promptly introduce and strengthen this already available option. Further local actions are promptly needed, above all in Lombardy where the recent decades have seen the weakening of the patient-centered medical home for the benefit of the hospital-centred approach, not enough in case of epidemic.

Rudan [1] well described the cascade of causes that dramatically led to COVID-19 tragedy in Italy. He described six main factors: 1) “premature relaxation around the real danger in Europe” even if this COVID-19 virus was an unknown one; 2) the possibility that “Chinese tourists form Wuhan were visiting Northern Italy in January and February 2020” with many possible asymptomatic transmissions; 3) the fact that “infected people from northern Italy spent their weekends at European ski resorts;” 4) the “biological bomb” of the Champions League football match between Atalanta (with more than 45 000 fans) and Valencia played in the San Siro stadium in Milan; 5) the spread of the virus in hospitals and retirements homes affecting many old patients; 6) “the omission to monitor the mathematical parameters of the epidemic, or perhaps the lack of clear communication of the dangers, or the indecisiveness to adopt isolation measures for the population.”

Rudan has completely explained the first causes and factors that generated a so negative outbreak in Italy. But in order to better explain a so critical situation, above all in Lombardy, it is necessary to focus on the factors of worsening that have arrested the functional Italian health system reply to the epidemic. Goumenou et al. [2] noted that administrative issues too created some problems: particularly the collapse of the health system was one of the most important one: “the lesson learned in the devastated Northern of Italy is that the infection spread should be controlled before the healthcare system reaches the saturation point,” avoiding that all the beds available in the health care facilities could have been occupied. In another recent paper Remuzzi et al. [3] described the critical situations in Italy and Lombardy providing urgent and useful indications to our national health systems and government at the regional level. The Italian Health System is quickly empowering the capacity of specific COVID-19 hospital admission (creating new COVID-19 emergency centres-hubs and new intensive care beds in the already existing hospitals) showing a great response in the first-line hospital-based treatment. But in order to functionally fight the COVID-19 pandemic in Italy and Lombardy, it is necessary to include the technology-based second-line: the urgent need of home-based remote monitoring systems could avoid the collapse of the hospital-centred first line, above all in Lombardy.

The major aim of this viewpoint is to underline the underestimated role of second line home-based treatment (for positive or suspected patients isolated at home) that could avoid to overwhelm the first line, taking into account the contribution of the necessary social limitations-containment campaign. Particularly the condition of acute respiratory failure could be promptly detected for COVID-19 patients in home isolation without not necessary and potentially dangerous hospital accesses and checks, but using easy-to-use (also for elderly patients and caregivers), comfortable, low-cost, reliable remote monitoring devices, such as connected pulse oximeters that can send real-time vital signs and other key biomedical parameters (especially respiration and blood oxygen levels, body temperature and other parameters) to a hospital-based big data server-repository, where Artificial Intelligence algorithms-based interpretation systems can elaborate these clinical records alerting the physicians only when it is necessary (not too early and not too late) to evaluate if moving a patient from a second to a first line. The experience of biomedical engineering and informatics, strong in chronic care management [4], could functionally help clinicians in managing many patients and citizens during this strong and long pandemic.

**Figure:**
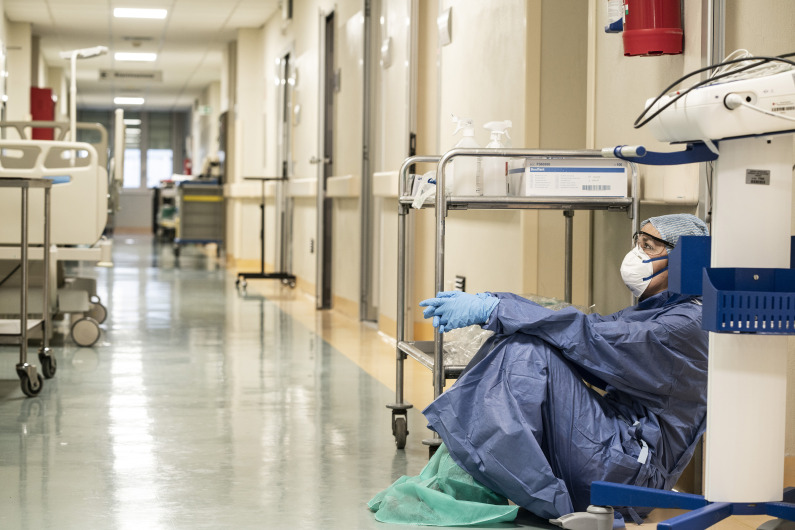
Photo: Doctor Annalisa Silvestri during COVID-19 pandemic 2020 in Italy, by Alberto Giuliani (CC BY-SA, https://creativecommons.org/licenses/by-sa/4.0).

Unfortunately experiences of telemedicine and telemonitoring in Italy are anecdotal, limited to local projects and not generalizable practices. According to Zanaboni and Lettieri [5], the implementation process of telemedicine requires a detailed sequence of steps: proofs of technology, pilot studies, clinical studies, large-scale services, institutionalized health-services. In Italy we have performed a lot of technologies and pilot studies, without moving temporary and not integrated telemedicine local projects to regional or national best practices that should have been introduced in the NHS-based clinical protocols and procedures.

It is not possible to postpone the implementation of large remote monitoring programs providing digital devices centrally controlled. We hope that Italian and International scientific and clinical community could promptly introduce and strengthen this already available option, as suggested by different Chinese associations of Medicine [6] and according to some emerging examples of applications in USA [7], where patients identified as likely positive for COVID-19 have not necessary to be admitted in an emergency department, but they can be monitored and treated at home with a thermometer and pulse oximeter to check their symptoms under the supervision of the health system's telehealth team (the so called “virtual hospital”). Taking into account that these patients could decompensate rapidly, a reliable and continuous monitoring system at home is an added value in the COVID-19 fight: many health care professionals could leave patients at home avoiding to admit them for observation in hospitals where acute care units are limited.

About the virtual patient monitoring approach something is moving also in Italy at national level [8] and local level, where the creation of the USCA (Unità speciali di continuità assistenziale – Special units of remote care) were declared for Lombardy on the 23^rd^ of March [9], allowing clinicians to include telemonitoring systems. Even if this project of quick and prompt telemonitoring program started, after more than one month (April 27, 2020) the USCA mhealth-based units were too few [10] and the hospital-based infections were still worrying. At the current time (June 29, 2020) the project for the achievement of all the USCA units in Lombardy is not yet completed and it is scheduled for the end of July 2020 [11].

Even if Italy has demonstrated many local and national technological actions (such as printing masks using 3D printers), a significant step forward is requested in order to develop a more coordinated general strategy to implement technologies into health care systems, also developing local and national clusters that could test and deliver biomedical devices and m-health solutions [12,13]. Only an approach coordinated by the political and administrative stakeholders could overcome the governmental lack of systematic integration of telemedicine and of home-based monitoring into the traditional hospital-based treatments.

Further local actions are promptly needed, above all in Lombardy where the recent decades have seen the weakening of the patient-centered medical home systems for the benefit of the hospital-centred approach, not the best one in case of epidemic. In Italy the telecare approach has to promptly move from an episodic and unstructured approach to an integrated and permanent key element of the general health care strategy, above all in chronic care management [4] and outbreak response. Telecare could minimize the risks to health care workers and patients reducing the burden during the COVID-19 pandemic. Italy and particularly Lombardy have to make policy changes to promote telecare use, equipping virtual hospitals and expanding programs and training for health care professionals. We need to learn from telemedicine failures developing functional examples for the future health care national and international systems. The fight against COVID-19 is still on and this crisis could be a great opportunity to solve old problems present in the Health Care Systems of Italy and Lombardy.
